# Comparative Analysis of the YABBY Gene Family of *Bienertia sinuspersici*, a Single-Cell C_4_ Plant

**DOI:** 10.3390/plants8120536

**Published:** 2019-11-22

**Authors:** Prabhakaran Soundararajan, So Youn Won, Dong Suk Park, Yeon-Hee Lee, Jung Sun Kim

**Affiliations:** Department of Agricultural Biotechnology, National Institute of Agricultural Sciences, Rural Development Administration, Jeonju 54874, Korea; prabhu89@korea.kr (P.S.); soyounwon@korea.kr (S.Y.W.); dspark@korea.kr (D.S.P.); yhl2222@korea.kr (Y.-H.L.)

**Keywords:** Genome sequencing, Whole genome duplication (WGD), YABBY gene family (YGF), Bienertia sinuspersici, diversification

## Abstract

The emergence and expression of the YABBY gene family (YGF) coincided with the evolution of leaves in seed plants, and was integral to the early evidence of lamina followed by reproductive development. YGF contains six subclasses, i.e., CRC, INO, FIL, YAB2, YAB3, and YAB5. This study aims to extract the genome sequences of the YGF in *Bienertia sinuspersici*, an important model plant for single-cell C_4_ (SCC_4_), non-Kranz photosynthesis. A comparative genomic analysis was undertaken with *Vitis vinefera*, *Arabidopsis thaliana*, *Brassica rapa*, and *Chenopodium quinoa*. Six copies of YGF were present in *B. sinuspersici* and *A. thaliana* with a single copy of each YGF subgroup. *V. vinefera* possessed seven copies of YGF with duplicates in FIL and YAB2 subgroups, but no YAB3. *B. rapa* and *C. quinoa* after whole genome duplication contained additional copies of YGF. The gene structure and conserved motifs were analyzed among the YGF. In addition, the relative quantification of YGF was analyzed in the leaves, reproductive developmental stages such as the bud, and the pre-anthesis and anthesis stages in *B. sinuspersici*, *A. thaliana*, and *B. rapa*. *CRC* and *INO* possessed conserved floral-specific expression. Temporal and perpetual changes in the expression of *YGF* orthologs were observed in the leaves and reproductive developmental stages. The results of this study provide an overview of YGF evolution, copy number, and its differential expression in *B. sinuspersici*. Further studies are required to shed light on the roles of YABBY genes in the evolution of SCC_4_ plants and their distinct physiologies.

## 1. Introduction

The YABBY gene family (YGF) is a class of transcription factors containing two conserved domains including a C2–C2 zinc-finger domain in the N-terminal and a high mobility group box in the C-terminal region. The high mobility group is also termed as “YABBY domain”, and consists of a helix–loop–helix motif [1,2,3]. YGF plays an essential role in the reproductive and vegetative systems of plants. The evolution of YGF is correlated with the origin of leaves in seed plants [4,5]. YGF is critical for the reorganization of the three-dimensional shoot model into flat appendages. Further changes occurred in juxtaposition with a unified abaxial–adaxial polarity and lamina maintenance [3,5,6,7,8,9]. A previous study of the basal eudicot *Eschscholzia californica* suggested that the role of YGF in converting shoots into bilateral leaves could have evolved before the early divergence of core eudicots [10]. Recently, Finet et al. (2016) described that one or two YGF could have existed in the last common ancestor before the diversification of gymnosperms and angiosperms [11]. In *A. thaliana*, YABBY gene family members were reported to be consisted with six subclasses, i.e., CRABS CLAW (CRC), INNER NO OUTER (INO), FILAMENTOUS FLOWER (FIL), YABBY2 (YAB2), YAB3, and YAB5 [3,10,11]. Generally, *CRC* and *INO* are called the “floral-specific YABBY genes” whereas *FIL*, *YAB2*, *YAB3*, and *YAB5* are referred to as the “vegetative YABBY genes” [5]. In general, the expression of the former two subgroups (*CRC* and *INO*) is restricted to the reproductive organs, whereas the latter four subgroups (*FIL*, *YAB2*, *YAB3*, and *YAB5*) are expressed in both the leaves and flower tissues [5,10].

In most eudicot primordia, the asymmetric expression of YABBY is restricted to the abaxial region [2,8,9] whereas expression has been detected as being adaxial-specific during the leaf initiation stage in *Zea mays* [8]. Even though the *CRC* genes are limited to the carpels and nectarines in eudicots [9], *CRC* homologs of rice and maize are involved in the midrib development of leaves [12,13]. The function of *INO* has been attributed to the abaxial identity of the outer integument of the ovules [7]. Apart from the abaxial cell fate and morphogenesis, the involvement of YABBY genes in abiotic tolerance such as drought, salt, and ABA stress were identified in *Glycine max* [14]. The negative regulation of YABBY genes under drought and salinity has been reported in *Gossypium hirsutum* [15]. FIL-like YABBYs in rice have been shown to be involved in the maintenance of meristem function [16,17]. Previous studies have shown that *FIL*, *YAB2*, and *YAB3* are associated with the abaxial domains of leaf-derived organs such as cotyledons, leaves, and floral organs [2,3,5,18]. *Oryza sativa* YABBY4 (OsYAB4), which is classified as the FIL/YAB3 subgroup, could have also been involved in the vasculature of rice, as it was predominant in the phloem tissue [19]. In tomato, *FAS*, which is a paralog to *YAB2*, was critical for fruit size and shape during domestication [20]. As YGF is closely associated with the emergence of flat-shaped leaves, and adapts diverse functions among plant species, it is considered an important evolutionary participant for the morphological diversification and regulatory processes [21]. Therefore, it is necessary to understand YGF evolution, copy number, and functional variation following duplication among lineages.

*Bienertia sinuspersici* is an important model plant for single-cell C_4_ (SCC4) photosynthesis [22]. *B. sinuspersici* lacks both mesophyll and bundle sheath cells, but still accomplishes C_4_ photosynthesis with dimorphic chloroplasts (non-Kranz anatomy) [23]. Subcellular compartmentalization of peripheral compartment chloroplast and central compartment chloroplast within single chlorenchyma cells are utilized for carbon fixation [24]. The adaptation strategy of *B. sinuspersici* and its anatomical features such as succulent leaves, rudimentary stigma, inconspicuous styles, and the tapering of stamen and pistil have created interest in studying the YABBY gene in vegetative and reproductive organs. Evolutionary studies on gene families in model and non-model plants have deepened our understanding of plant genome architecture. Most modern plants contain evidence of ancient polyploidization, such as paleohexaploidy (γ) and paleotetraploidy (β and α) events [25]. Through recent whole genome triplication (WGT), it has been shown that three paralogous subgenomes of *Brassica rapa* were generated 13 to 17 million years ago (MYA), very soon after the *Arabidopsis* and *Brassica* divergence occurred at 17 to 18 MYA [26]. *Chenopodium quinoa* (2n = 4x = 36) is a tetraploid which recently hybridized (3.3–6.3 MYA) from its diploid progenitors, *Chenopodium pallidicaule* (A-genome) and *C. suecicum* (B-genome) [27]. Phylogenetically, *B. sinuspersici* is more closely related to *C. quinoa* than it is to the other species. *Vitis vinefera* is an outgroup for eudicots that diverged approximately 130–240 MYA from monocotyledons. As the grapevine genome has not undergone recent genome duplication and contributes ancestral chromosomes to all rosids (2n = 38) [28], it has been used to determine the genetic organizational features of YGF after whole genome duplication (WGD). The evolution of polyploidy involved a highly complex and dynamic process compared to that of diploids. Drastic genomic reorganization, genetic and epigenetic modifications, and functional adaptations restore diploid-like behavior in many polyploid genomes. The shuffling of whole chromosomes leads to the diploidization behavior of old (e.g., *B. rapa*) and recent allopolyploids (e.g., *C. quinoa*) [29].

Although YGF have been reported as being evolutionarily integral to the genetic reprogramming of ancestral shoots into leaf-specificity and as constitutive factors for the floral morphogenesis of seed plants, no previous studies have examined YABBY in SCC_4_ plants. Therefore, the primary objective of this research was to identify and characterize YABBY genes in *B. sinuspersici*. Further comparative genomic studies were undertaken to determine the evolutionary and functional changes that YGF underwent during past and recent WGD.

## 2. Results

### 2.1. Identification, Classification, and Phylogenetic Tree Analysis of YABBY Genes

**Figure 1:**
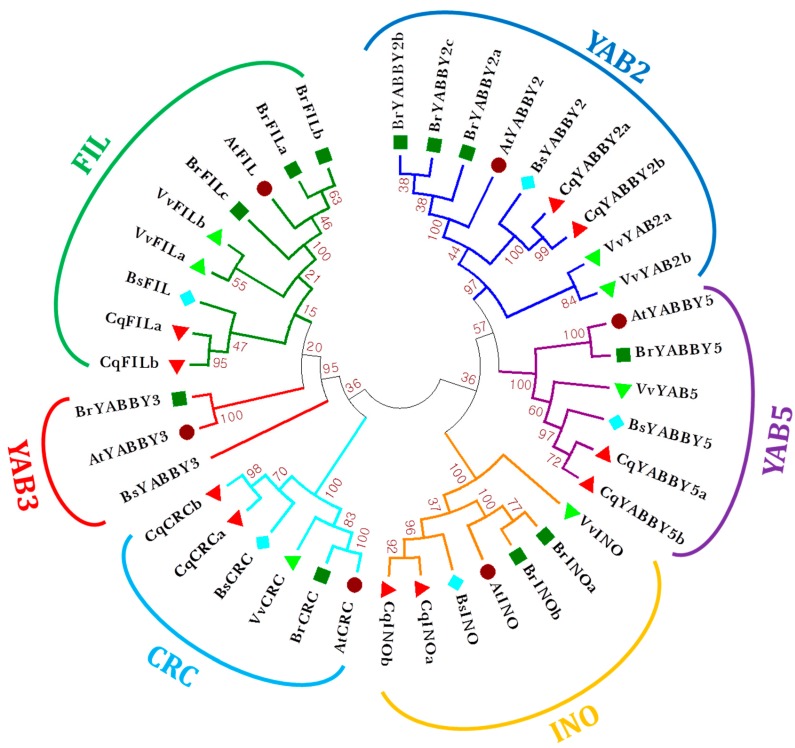
Phylogenetic tree of the YABBY gene family in *Vitis vinefera*, *Arabidopsis thaliana*, *Brassica rapa*, *Bienertia sinuspersici*, and *Chenopodium quinoa* constructed by the maximum likelihood method using MEGA v. 6.0. Light green down triangle, *V. vinefera*; Brown circle, *A. thaliana*; Dark green square, *B. rapa*; Blue diamond, *B. sinuspersici*; and Red up triangle, *C. quinoa*.

*A. thaliana* (*At*) and *B. sinuspersici* (*Bs*) have six copies of YGF, and exist as single copy subgroups. For the identification of YABBY genes in *B. sinuspersici*, we used its whole genome protein sequences. De novo genome sequencing of *B. sinuspersici* is currently at the completion stage (Kim et al. Unpublished). *V. vinefera* possesses seven copies of YGF with duplicates in the FIL and YABBY (YAB) subgroups with YAB3 missing. Some of the YABBY gene subgroups are present in multiple copies in *B. rapa* (*Br*) and *C. quinoa* (*Cq*) (Figure 1). A total of 11 and 10 YABBY genes were identified in *B. rapa* and *C. quinoa*, respectively. Two orthologs of INO and three orthologs of YAB2 and YAB3 were present in *B. rapa*, while the copy numbers of *CRC*, *FIL*, and *YAB5* remained as a single copy. With the absence of YAB3, the other subgroups of the YABBY genes were present as two copies in *C. quinoa*. Most importantly, a phylogenetic tree showed that the clade with CRC and FIL/YAB3 cluster was separated and distinguished from the INO, YAB2, and YAB5. Genomic information about the YGF and their genomic positions are shown in Table 1. The exon-intron structures of the YGF are given in Figure S1. Complete CDS sequences of *B. sinuspersici* YGF are provided in the Supplementary data.

### 2.2. MOTIF and Functional Domain Analysis of YABBY Gene Family Members

**Figure 2:**
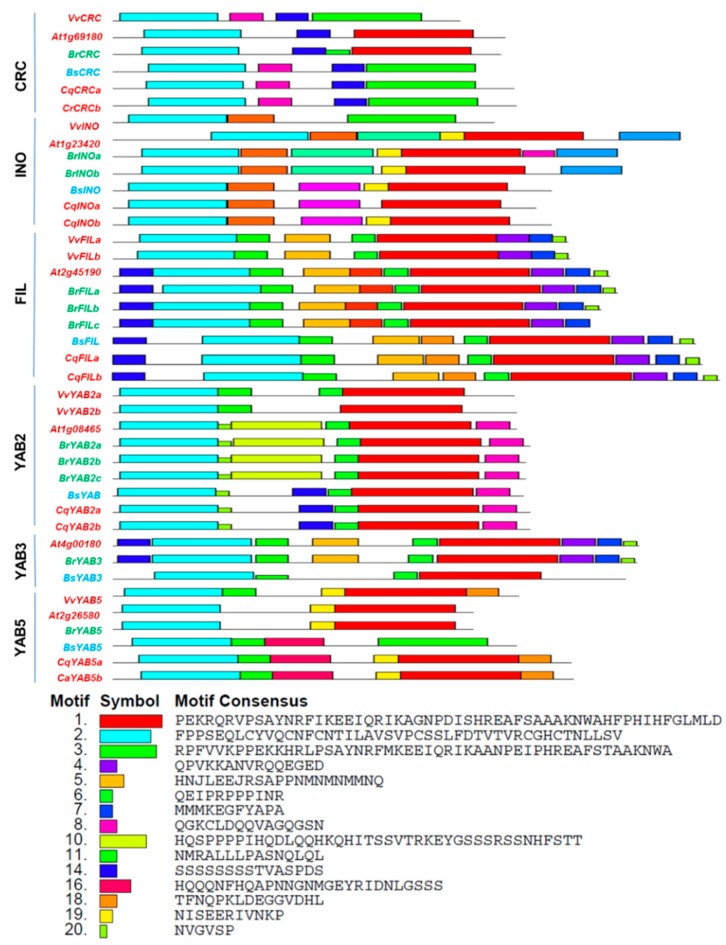
Motif of the YABBY gene family in *Vitis vinefera*, *Arabidopsis thaliana*, *Brassica rapa*, *Bienertia sinuspersici*, and *Chenopodium quinoa*, identified using the MULTIPLE EM for MOTIF ELICITATION (MEME) server. Details of the motifs are shown in Table S1. Motif numbers.

The conserved motifs investigated in YGF between the species are shown in Figure 2 and Table S1. Unlike the N-terminal C2–C2 zinc-finger domain, a change in amino acids was observed on the YABBY domain in VvCRC, BsCRC, CqCRCa, CqCRCb, VvINO, and BsYAB5 (Figure S2). The CRC subgroup shares motif 14 with FIL + YAB3 and YAB2. INO displayed four phenomena, i.e., being collectively present in all plant species (motif 9), except outgroup (motif 19), only within Brassicales (motifs 12 and 13), and unique to Caryophyllales (motif 15). Similarly, the vegetative specific motifs were observed in YAB2 (motif 10) and FIL (motif 17), and YAB5 (motif 16) for Brassicales and Caryophyllales, respectively. Aside from the two functional domains, FIL + YAB3 were recognized with seven conserved motifs either alone or with other subgroups. In particular, motifs 4, 5 and 7, that were exclusive to FIL and YAB3 in all species, were excluded in BsYAB3. Similarly, motifs 6 and 20 between FIL + YAB3 and YAB2, and motif 14 with CRC, FIL + YAB3, and YAB2, were not observed in BsYAB3. BsYAB3 shared motif 11 with all vegetative YABBY subgroups. Nonetheless, motif 18 was noted only in the Vv, Bs, and Cq subgroups. At CRC, VvYAB2, AtYAB5, and BrYAB5 were characterized with fewer motifs.

### 2.3. Expression of YGF in Vegetative and Reproductive Tissues

**Figure 3:**
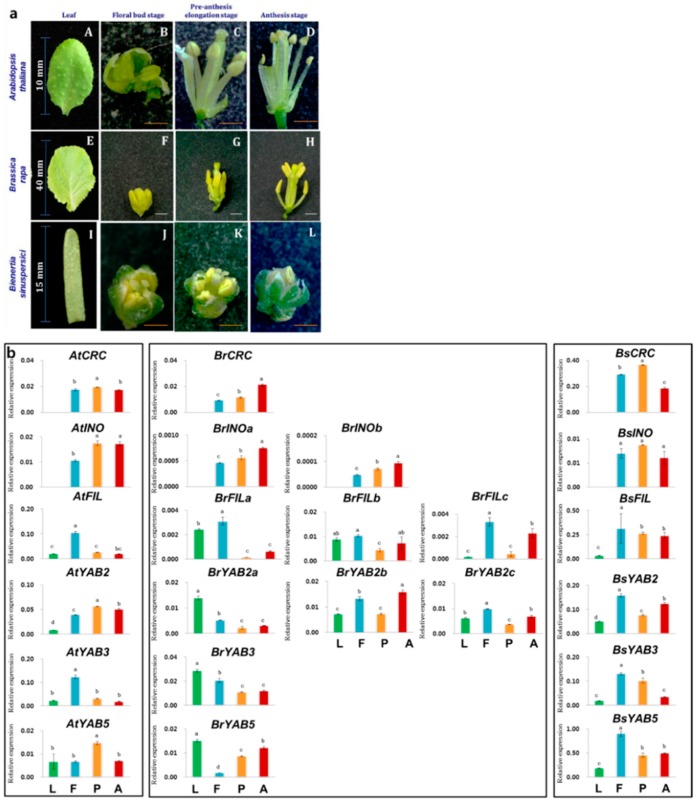
(**a**) Leaf and reproductive developmental stages of *Arabidopsis thaliana* (A-D), *Brassica rapa* (E-H), and *Bienertia sinuspersici* (I-L). The floral stage images were taken using a microscope (www.dimis.co.kr). Orange bar, 100×; White bar, 50×. (**b**) Real-time quantitative-polymerase chain reaction analysis of YABBY gene expression in *Arabidopsis thaliana* (*At*), *Brassica rapa* (*Br*), and *Bienertia sinuspersici* (*Bs*). Legends: L, Leaf; F, Floral bud stage; P, Pre-anthesis elongation stage; and A, Anthesis stage. Bars indicate mean ± standard error.

We examined YGF expression in the leaves and different reproductive developmental stages such as the bud, pre-anthesis, and anthesis in *A. thaliana*, *B. rapa*, and *B. sinuspersici* (Figure 3a). The expression of *CRC* and *INO* was restricted to the reproductive organs in all three species. Both *BsCRC* and *BsINO* expression progressively increased from the bud to the anthesis stages. The expression of *AtINO* was lower in the bud stage than in the pre-anthesis and anthesis stages. The highest expression levels of *BsCRC* and *BsINO* were observed in the pre-anthesis stage, and both genes were downregulated in the anthesis phase. *BrCRC* and *BrINO* in *B. rapa* also showed an increased expression during the reproductive organ developmental stages. *AtFIL* and *AtYAB3* had similar expression patterns in the four different tissues as the upregulation of *AtFIL* and *AtYAB3* during the floral bud stage. High expression of *AtYAB2* and *AtYAB5* was observed during the pre-anthesis stage. The paralog copies of *BrFIL* and *BrYAB2* were upregulated during the floral bud stage compared with the pre-anthesis elongation stage. Except for *BrCRC*, *BrINO*, and *BrYAB5*, the *BrYABBY* genes were less expressed during the pre-anthesis elongation stage compared to the other reproductive developmental stages. *BrYAB2a*, *BrYAB3*, and *BrYAB5* genes had higher expressions in the leaf tissue. *BsFIL*, *BsYAB2*, *BsYAB3*, and *BsYAB5* were expressed less in the leaves than in the floral tissues of *B. sinuspersici*, which was similar to that found in *A. thaliana* (except for *AtYAB5*). Among the reproductive stages, the floral bud phase showed upregulation of *BsFIL*, *BsYAB2*, *BsYAB3*, and *BsYAB5*. Compared with other reproductive developmental stages, *BsYAB2* and *BsYAB5* were downregulated during the pre-anthesis elongation stage (Figure 3b).

## 3. Discussion

YABBY genes are essential key regulators that are involved in the evolution of leaves in seed plants. They govern various developmental processes such as shoot apical meristem restriction, polarity, laminar development, establishment of leaf margin, floral differentiation, carpel morphogenesis, outer integument growth, nectarines, and inflorescence, as well as other regulatory and development processes [2,5,8,10,15,30].

One or two copies of YABBY gene(s) in the last common ancestor of gymnosperms and angiosperms split into five subgroups in the basal eudicot [10,11]. *V. vinifera* is the first plant genome to provide unexpected evidence for genome duplication events in species that had previously been considered as true diploids based on their genetics [28]. However, the grape possesses seven copies, which suggests that the ancient polyploidization and retention of higher copy number could have contributed to the abundance of duplicate genes in YGF [31]. Polyploidization could have been a driving force for acquiring more copies of gene families [32]. Genes evoked from polyploidization are usually removed by following small-scale duplication and rearrangement [33]. Despite having different genome sizes, *A. thaliana* (approximately 135 Mb) and *B. sinuspersici* (anticipated genome size of 3.8 Gb, unpublished data; genome assembly of *B. sinuspersici* have been performed using long-read sequencing by Kim et al. team) both retained a single copy of YGF in each subfamily. Proceeding recent duplication and angiosperm diversification produces several copies of YABBY genes among plant species [28]. Mesohexaplodization of *B. rapa* [25] and tetraploidization of *C. quinoa* [27] have created an expansion of the subsets of YGF members (Figure 1). Based on evolutionary intricacies and complexities, many orthologous copies of YABBY subfamilies exist and vary between species (Table 1). CRC and FIL+YAB3 are monophyletic clades having unique synapomorphies [10,11]. Our phylogenetic analysis (Figure 1) supports a common ancestor clade that could be composed of CRC and FIL monophyletic, followed by the paraphyletic evolution of other YABBY subfamilies [10]. Similar to *A. thaliana*, *B. rapa*, and *B. sinuspersici*, plant species such as *Gossypium hirsutum*, *Z. mays*, and *Glycine max* also possess a single copy of *CRC* [14,15]. In contrast, two copies of *CRC* were present in *C. quinoa*, probably because of the recent WGD that occurred approximately 3.3–6.3 MYA [27]. *FIL* and *YAB3* could have formed when an *At*-α duplication occurred approximately 14.5–86 MYA, as both share the same clade due to high homology and are also not well diverged in *V. vinifera* [34]. A complex WGT process (13–17 MYA) created the existence of three copies of *BrYAB2*. The hybridization of diploid progenitors produced two copies of *CqYAB5*. This major increase in the genome was succeeded by massive subgenome relocalization, chromatid recombination, and fractionation [35]. The usual fate of duplicate genes is conversion back to a single copy. The retention of duplicate genes generated from polyploidization is genuinely evolutionary, as it contributes to potential adaptation. The genes involved in signal transductions and transcriptions are preferentially retained after WGD [32,33]. Differences in retention rates in plants that experience WGD/WGT depend on the functional efficiency and fine-tuning of gene regulatory networks. The components of multi-subunit complexes have to be in stoichiometric balance to properly function [32]. A maintained copy number with two paralogs of *VvFIL* and *VvYAB2* and three paralogs of *BrFIL* and *BrYAB2* were found in each genome. The lack of duplicates in the *BrCRC*, *BrYAB3*, *BrYAB5*, *CqFIL*, and *CqYAB3* subgroups occurred because additional copies must be deleted; otherwise they are deleterious [10]. Nevertheless, the counter-balance among the genome rearrangement, unequal chromosomal crossover, and retainment of relaxed selection determined the gene dosage balance model [25,35,36,37]. The variations in the exon-intron arrangement (Table 1, Figure S1) demonstrated the structural adaptation experienced by the YABBY genes corresponding with an evolutionary timescale. Introns are important factors in the determination of the functional divergence of proteins via exon shuffling and differential splicing [38]. The observation that one or two exon(s) absent in the *CRC* and *INO* subgroups of *Vv*, *Bs*, and *Cq*, and the YAB5 of *At*, *Br*, and *Bs* are correlated with a reduction in protein length (Table 1) provides the information that without bias, the loss of exons occurred in the genomes of different lineages.

A shift in amino acids on the YABBY domain (Figure S1) in a few clades was conjoint with changes in the carpel development pattern across angiosperms. CRC motifs are not shared with INO motifs, which indicates that these groups are polyphyletic with unique synapomorphy. INO functions are restricted to the outer integument of the ovule and its outgrowth [5]. Four types of phenomena were observed in INO motifs (Table S1) resembling the process of integumentary variation invoked among clades. The common conserved motif (9) was marked with the retention of the gametophyte. Caryophyllales specific to the INO motifs existed in both *C. quinoa* and *B. sinuspersici*, and increased in *BsINO*, as both have apparent variation in seed formation. Future studies should be conducted to clarify whether developmental retardation or congenital integument fusion [39] is occurring in *B. sinuspersici*. The higher number of common motifs detected between FIL and YAB3 represented functional overlap and recent duplication (Figure 2). Apart from the presence of common motif 11 conserved with other plant species, the absence of conserved motifs such as 4, 5, 6, 7, and 20 in BsYAB3 provided evidence of the functional transit in leaf differentiation of *B. sinuspersici*. The double mutation of *FIL* and *YAB3* in *A. thaliana* failed on leaf primordia differentiation and polarity initiation [5]. The conserved motifs not discovered in BsYAB3 were presumed to be the evolutionary diversification of *B. sinuspersici*. Sequencing-based annotation phenomena showed reasonable variation, and further experimental studies must be conducted to confirm the distinct *BsINO*, together with *BsYAB3* and its integral part in the *B. sinuspersici* phenotype.

*CRC* and *INO* were uniformly expressed only in the reproductive developmental stages of *A. thaliana*, *B. rapa*, and *B. sinuspersici*, suggesting their conserved function in carpel morphogenesis, gynoecium differentiation, floral meristem, and outer integument development [21,40]. High *BsCRC* expression during the pre-anthesis elongation stage was detected. These patterns of high pre-anthesis elongation stage and decreases during the anthesis stage have been reported in *Solanum lycopersicum* L. (*Sl*) *SlYAB2*, *SlCRC*, and *SlINO* low-locule-number tomato (small) cultivar [41]. Therefore, downregulation of *CRC* and *INO* from the pre-anthesis stage to the anthesis stage in *B. sinuspersici*, unlike that in *A. thaliana* and *B. rapa*, but the same in the small tomato, reflects species/lineage-specific or different fruit formation expression. The progressive expression patterns observed in *A. thaliana* and *B. rapa (*Figure 3b) displayed the importance of *INO* in determining the outer integument growth of the ovule [42]. The analogous expression of *AtFIL* and *AtYAB3* denotes their identical function on axial patterning, shoot meristem identity, lateral organ development, and phyllotaxy of inflorescence [43]. The considerable differential expression patterns of *FIL*, *YAB2*, and *YAB5* orthologous genes demonstrated their species-specific, non-autonomous role in organogenesis [15,44,45]. Nevertheless, the *YAB3* gene was homogeneously downregulated from the bud to the anthesis stage in all three species, illustrating its importance in the early stages of floral meristem establishment and the differentiation of inflorescence in diversified plants [3,4,5,30]. Other than the higher expression of *BrFILb* and *BrFILc* in the anthesis stage than that in the pre-anthesis stage, almost a uniform framework was maintained on FIL among the species (Figure 3b). Finally, relative quantification analysis suggested that among the YABBY subgroups, *CRC*, *INO*, *FIL*, and *YAB3* maintained some conserved function, while differential expression denotes either a temporal or perpetual shift in functional divergence. Spatial and temporal expression pattern of YABBY genes has been reported in rice along with other transcription factors during embryogenesis [46]. Similarly, leaf and floral organ primordia showed spatial and temporal expression of *FIL* in *A. thaliana* [18]. Lower expression of YABBY genes in leaf tissue of *A. thaliana* and *B. sinuspersici* was observed compared to *B. rapa*. Regarding our result of *B. rapa*, in tomato, the expression of *SlYAB3* and *SlYAB5b* in leaves were higher than the flowers [41]. Though in this study we haven’t focused on photosynthesis, it is interesting to note that in *A. thaliana*, C_3_ model plant YABBY genes are expressed in abaxial cells, whereas in *Z. mays* expression is observed in adaxial cells [3,8]. Since *B. sinuspersici* showed variation in the expression with the model plant *A. thaliana* and crop *B. rapa*, future work on YABBY genes focusing on the C_3_ and C_4_ mechanisms could provide more knowledge on the key genes involved in structural changes to support the partitioning and the polarity of chloroplast in central and peripheral regions within single chlorenchyma cells.

## 4. Materials and Methods

### 4.1. Identification of the YABBY Gene Family in Arabidopsis thaliana, Brassica rapa, Bienertia sinuspersici, and Chenopodium quinoa

The protein sequences of *V. vinifera* (https://phytozome.jgi.doe.gov/pz/portal.html#, Genoscope.*12X*), *A. thaliana* (http://plants.ensembl.org/, TAIR10, INSDC Assembly GCA_000001735.1), *B. rapa* (Assembly Brapa_1.0, ID: 229), *C. quinoa* (Assembly ASM168347v1, ID: 12754, https://www.ncbi.nlm.nih.gov/), and *B. sinuspersici* (Iso-seq data using PacBio technique, unpublished by Jung Sun Kim) were subjected to the hidden Markov model (HMM) [47] analysis with the YABBY domain seed file (PF04690) [48] at an E value of 10^−5^. In the Brassica database (BRAD; http://brassicadb.org/brad/), several releases of the reference *B. rapa* genome (v 1.5 [25], v 2.5 [49], and v 3.0 [50]) had an ambiguous interpretation of the YGF. Additionally, the well-characterized *A. thaliana* YABBY genes were BLAST against the *V. vinifera*, *B. rapa*, *C. quinoa*, and *B. sinuspersici* genomes. The results of both HMM and BLAST were compared, and repeats were removed manually by self-BLASTp. The truncated peptides and proteins having the same chromosomal position were also eliminated. Furthermore, a protein family domain analysis was manually conducted on the identified protein sequences to confirm the presence of the C2–C2 zinc-finger-like and YABBY domains. The protein sequences without functional domains were removed, and the YABBY genes were confirmed in each plant species.

### 4.2. Phylogenetic Tree Construction

The finalized protein sequences were aligned in the MEGA v. 6.0 software using ClustalW. A phylogenetic tree was constructed using the maximum likelihood statistical method, 1000 bootstrap replications, partial deletion (95), and nearest-neighbor-interchange [51].

### 4.3. Exon-Intron Organization

The YABBY proteins were BLAST (tBLASTn) against contigs of *B. sinuspersici*. The contigs showing the highest homologies with low E values were extracted for the respective YABBY genes [52]. Protein-based gene predictions were performed with contigs to determine the genomic positions using the FGENESH + HMM profile with the dicot plants, using the *Arabidopsis*-specific option [53]. For *V. vinifera*, *A. thaliana*, *B. rapa*, and *C. quinoa*, genomic information for YABBY genes was retrieved from databases to determine gene structure. BED tools were used to extract the genomic positions of YABBY genes [54]. The coding sequence regions of the longest isoforms were chosen, and the exon-intron arrangements of the YGF members were analyzed using the Gene Structure Display Server (GSDS) v. 2.0 [55].

### 4.4. Conserved Motif Analysis

MEME (v. 4.9.1) was used to identify the conserved motifs of the YGF proteins [56]. The parameters set to determine the motifs were zero or one occurrence and 20 motifs, with a minimum width of six and a maximum width of 200. The functional amino acid regions encoding the C2–C2 zinc-finger-like domain and the YABBY domain were identified with the MEME server, and were separately retrieved and aligned using ClustalW to find the insertions/deletions within the functional domain of the YGF proteins.

### 4.5. RNA Isolation and Expression Analysis of YABBY Genes

Total RNA was isolated from the 100 mg of lyophilized leaves, floral bud stage, pre-anthesis stage, and anthesis stage of greenhouse grown *A. thaliana* (Columbia ecotype), *B. rapa* spp. *perkinensis* (Chiifu 401–42 line), and *B. sinuspersici* (BioSample SAMN03290884) using the RNeasy^®^ Plant Mini Kit (Qiagen, Germany) following the manufacturer’s instructions. Before homogenization with RLT buffer, samples were finely ground using TissueLyser II (Qiagen, Hilden, Germany) without allowing them to thaw. DNase I (Recombinant DNase I, Takara, Shiga, Japan)-treated RNA (2 µg) was subjected to cDNA synthesis (AmfiRivert II cDNA Synthesis Master Mix, GenDEOPT, Massachusetts, USA). Real-time quantitative-polymerase chain reaction (RT-qPCR) (iQ^TM^ SYBR Green^®^ Supermix, Biorad, California, USA) was conducted with a 10-fold dilution of cDNA. Glyceraldehyde-3-phosphate dehydrogenase (*GAPDH*) was used as an internal control for the relative expression analysis of YABBY genes. Primers with different nucleotides on their 3′ends were designed for *B. rapa* paralogs, which shared a high percentage of homology (Table S2). The thermocycler conditions (CFX96 Touch^TM^ Real-Time PCR Detection System, Biorad, California, USA) used for the RT-qPCR were the following: 95 °C for 3 min, 40 cycles of 95 °C for 15 s, 58 °C for 30 s, and 95 °C for 10 s; Melting curve: 95 °C for 10 s, 65 °C for 31 s, and 65 °C for 0.05 s, with + 0.5 °C/cycle and Ramp of 0.5 °C/s. Each reaction was performed in three biological replicates. Gene expression was normalized relative to *GAPDH* [57]. The expressions of *GAPDH* between different tissue types of *A. thaliana*, *B. rapa*, and *B. sinuspersici* are given in Table S3. One-way analysis of variance was conducted for statistical analysis with *P* < 0.05 using Duncan’s post-hoc test (SPSS for Windows v. 16.0 SPSS Inc., Chicago, USA).

## 5. Conclusions

YABBY family genes (YGF) determine important functions in the development of the leaf, flower, and fruit. The single orthologs of YABBY subgroups existed in *A. thaliana* and *B. sinuspersici*, and are present as seven copies in eudicot outgroup genome, *V. vinifera*. Because of polyploidization, the subgroup of YGF was expanded in *B. rapa* and *C. quinoa* to 11 and 10 copies, respectively. The differences in the number of YGF between plants species could be due to natural selection. The motif study revealed the fact that conserved, as well as differential, motifs exist between clades/species. For instance, the loss of the conserved motif on BsYAB3 would wreak havoc in a flat-shaped leaf architecture. The specificity of *CRC* and *INO* for the reproductive tissues is evidence of a conserved function. Nevertheless, the differential fashion of expression between tissue types and developmental stages was observed in all YABBY subgroups. From the studied genome, it is clear that YGF members vary between species. Differences in the number of YGF could be due to natural selection. The functional differentiation of genes is a common consequence of evolution. The overall findings suggest that YABBY could have unique functional adaptation in the leaf and floral organ development of *B. sinuspersici.*

More importantly, the expression of the cell fate of YABBY differed, as abaxial and adaxial in C_3_ (*A. thaliana*) and C_4_ (*Z. mays*) photosynthetic plants, respectively. Further studies on SCC_4_ photosynthesis could provide a useful framework in which to interpret the pattern of changes which occurred on vacuolar and cytoskeleton development to support the compartmentalization, anchoring, and polarity maintenance of central and peripheral chloroplast in *B. sinuspersici*.

## Figures and Tables

**Table 1:** Information of the YABBY gene family in Vitis vinefera, Arabidopsis thaliana, Brassica rapa, Bienertia sinuspersici, and Chenopodium quinoa.

*Sub Class Name*	*Species*	Gene ID	*Gene Name*	Genome Location	Genomic Position	Strand	Gene Length (bp)	Exon Count	Protein Length (Amino Acids)
*CRABS CLAW (CRC)*	*V. vinifera*	GSVIVT01012246001	*VvCRC*	Chr1	237202–239435	-	2257	6	163
	*A. thaliana*	NP_177078.1	*At1g69180*	Chr1	26007350–26009141	-	1792	7	181
	*B. rapa*	XP_009105464.1	*BrCRC*	A07	18254274–18255932	-	1659	7	179
	*B. sinuspersici*	MK782018	*BsCRC*	005453F^1^	51–2112	-	2177	5	167
	*C. quinoa*	XP_021729181.1	*CqCRCa*	US^2^	19585655–19589475	-	3821	6	185
	*C. quinoa*	XP_021717873.1	*CqCRCb*	US^2^	5881243–5884417	-	3175	6	186
*INNER NO OUTER (INO)*	*V. vinifera*	GSVIVT01013778001	*VvINO*	Chr1	7691531–7692677	+	1159	6	179
	*A. thaliana*	NP_001320962.1	*At1g23420*	Chr1	8317297–8319491	+	2195	7	262
	*B. rapa*	XP_009115528.1	*BrINOa*	A09	24145453–24147639	-	2187	7	233
	*B. rapa*	XP_009103178.1	*BrINOb*	A07	8139144–8141089	-	1946	7	235
	*B. sinuspersici*	MK782019	*BsINO*	001733F^1^	420625–422407	+	2193	6	202
	*C. quinoa*	XP_021742940.1	*CqINOa*	US^2^	1945675–1947825	-	2151	6	195
	*C. quinoa*	XP_021727415.1	*CqINOb*	US^2^	3336213–3348004	-	11792	6	202
*FILAMENTOUS FLOWER (FIL)*	*V. vinifera*	GSVIVT01027648001	*VvFILa*	Chr15	14674818–14677943	+	3158	7	214
	*V. vinifera*	GSVIVT01001269001	*VvFILb*	Chr2	4861965–4864774	+	2839	7	215
	*A. thaliana*	NP_566037.1	*At2g45190*	Chr2	18628252–18630779	-	2528	7	229
	*B. rapa*	XP_009142321.1	*BrFILa*	A04	18508497–18511151	-	2655	7	233
	*B. rapa*	XP_009133711.1	*BrFILb*	A03	10826573–10829091	-	2519	7	225
	*B. rapa*	XP_009104094.1	*BrFILc*	A07	12540446–12549261	+	8816	7	220
	*B. sinuspersici*	MK782020	*BsFIL*	001741F^1^	386662–391675	+	5973	7	269
	*C. quinoa*	XP_021748608.1	*CqFILa*	US^2^	598260–602286	-	4027	7	269
	*C. quinoa*	XP_021756313.1	*CqFILb*	US^2^	728094–732187	-	4094	7	277
*YABBY2*	*V. vinifera*	GSVIVT01037533001	*VvYAB2a*	Chr6	11951270–11958015	+	6814	6	187
	*V. vinifera*	GSVIVT01022586001	*VvYAB2b*	Chr8	5502500–5509539	+	7111	6	188
	*A. thaliana*	NP_001077490.1	*At1g08465*	Chr1	2675813–2679824	+	4012	6	184
	*B. rapa*	XP_009148101.1	*BrYAB2a*	A06	2922075–2926617	+	4543	6	190
	*B. rapa*	XP_009110891.1	*BrYAB2b*	A08	20641200–20645565	-	4366	6	188
	*B. rapa*	XP_009118372.1	*BrYAB2c*	A09	35529819–35533946	-	4128	6	188
	*B. sinuspersici*	MK782021	*BsYAB2*	001107F^1^	471182–481934	-	12601	6	187
	*C. quinoa*	XP_021720585.1	*CqYAB2a*	US^2^	6293447–6298238	-	4792	6	190
	*C. quinoa*	XP_021715646.1	*CqYAB2b*	US^2^	1999454–2003677	+	4224	6	190
*YABBY3*	*A. thaliana*	NP_567154.1	*At4g00180*	Chr4	72545–75576	-	3032	7	240
	*B. rapa*	XP_009111512.1	*BrYAB3*	A09	1090354–1093737	+	3384	7	239
	*B. sinuspersici*	MK782022	*BsYAB3*	014573F^1^	15118–20700	+	6638	7	234
*YABBY5*	*V. vinifera*	GSVIVT01015567001	*VvYAB5*	Chr11	5013887–5017668	+	3820	7	189
	*A. thaliana*	NP_850080.1	*At2g26580*	Chr2	11303455–11307010	-	3556	6	164
	*B. rapa*	XP_009133935.1	*BrYAB5*	A03	11646962–11651411	+	4450	6	164
	*B. sinuspersici*	MK782023	*BsYAB5*	006059F^1^	26927–30927	-	4643	6	184
	*C. quinoa*	XP_021741749.1	*CqYAB5a*	US^2^	3725859–3730115	-	4257	7	209
	*C. quinoa*	XP_021760782.1	*CqYAB5b*	US^2^	1054209–1058320	+	4112	7	210

F^1^ contigs number; US^2^, Unplaced Scaffold.

## Supplementary Materials

The following are available online at https://www.mdpi.com/2223-7747/8/12/536/s1, Figure S1: Gene structure of YABBY gene family in *Vitis vinefera*, *Arabidopsis thaliana*, *Brassica rapa*, *Bienertia sinuspersici*, and *Chenopodium quinoa* analyzed using GENE STUCTURE DISPLAY SERVER (GSDS) v. 2.0, Figure S2: Multiple sequence alignment of YABBY functional domains of *Vitis vinefera, Arabidopsis thaliana*, *Brassica rapa*, *Bienertia sinuspersici*, and *Chenopodium quinoa* align by ClustalW using MEGA v. 6.0. Table S1: Motifs identified in YABBY gene family of *Vitis vinefera*, *Arabidopsis thaliana*, *Brassica rapa*, *Bienertia sinuspersici*, and *Chenopodium quinoa*., Table S2: Primers used for RT-qPCR analysis, Table S3: Expression of *GAPDH* in threshold cycle (C_T_) value between different tissue types studied in *Arabidopsis thaliana* (*At*), *Brassica rapa* (*Br*), and *Bienertia sinuspersici* (*Bs*). Supplementary data: Complete CDS sequences of *B. sinuspersici* YGF.

## Abbreviations

AS1	ASYMMETRIC LEAVES1
*At*	*Arabidopsis thaliana*
ARF	AUXIN RESPONSE FACTOR
BRAD	Brassica database
*Br*	*Brassica rapa*
BOP	BLADE ON PERTIOLE
Bs	Bienertia sinuspersici
CRC	CRABS CLAW
Cq	*Chenopodium quinoa*
FIL	FILAMENTOUS FLOWER
GAPDH	Glyceraldehyde-3-phosphate dehydrogenase
HMM	Hidden Markov model
INO	INNER NO
LOB	LATERAL ORGAN BOUNDARY
MEME	Multiple em for motif elicitation
PHB	PHABULOSA
PHAN	PHANTASTICA
PHV	PHAVOLUTA
RS2	ROUGHSHEATH2
SCC_4_	single-cell C_4_
*Vv*	*Vitis vinefera*
YGF	YABBY gene family
WGD	Whole Genome Duplication
WGT	Whole Genome Triplication
