# Supplementary material for: Comparative Analysis of the YABBY Gene Family of Bienertia sinuspersici, a Single-Cell C4 Plant
Source: Plants (Basel). 2019 Nov 22;8(12):536. doi: 10.3390/plants8120536 (PMC6963775; doi:10.3390/plants8120536)
Supplement: Supplementary file 1 [file plants-08-00536-s001.zip › spm-plants-619033/Supplementary Table_1.docx]

**Supplemnet Table 1.** Motifs identified in YABBY gene family of *Vitis vinefera*, *Arabidopsis thaliana*, *Brassica rapa*, *Bienertia sinuspersici*, and *Chenopodium quinoa*.

| No | MOTIF pattern | Site | Width | YABBY group |
| --- | --- | --- | --- | --- |
| 1. | PEKRQRVPSAYNRFIK[ED]EIQRIKAGNP[DE]ISH[RK]EAFS[AT]AAKNWAHFPHIHFGLM[LP]D | 40 | 55 | All |
| 2. | F[PS][PS]S[ED][QHR][LV]CYV[QH]C[NS]FC[NT]T[IV]LAVSVP[CY][ST]S[LM]F[DK]TVTVRCGHCTNLLS[VL] | 34 | 45 | Except VvCRC, VvINO, BsCRC, BsYAB5, CqCRCa, CqCRCb. |
| 3. | RPFV[CNV]KPPEK[KR][HQ]RLPSAYNRF[MI]KEEIQRIKAANPEIPHREAFSTAAKN[WV]A | 6 | 50 | VvCRC, BsCRC, CqCRCa, CqCRCb |
|  |  |  |  | VvINO |
|  |  |  |  | BsYAB5 |
| 4. | QPVKK[AT]N[VM][RP]QQ[EQ][GE][EG][DE] | 11 | 15 | VvFILa, VvFILb, AtFIL, BrFILa, BrFILb, BsFILc, BsFIL, CqFILa, |
|  |  |  |  | AtYAB3, BrYAB3, CqYB3 |
| 5. | [HQ]N[LI]LEE[IL]R[SD][AS]P[SP]N[MI][NL][MI][NM][MN][MH][NH]Q | 11 | 21 | VvFILa, VvFILb, AtFIL, BrFILa, BrFILb, BrFILc, BsFIL, CqFILa |
|  |  |  |  | AtYAB3, BrYAB3, CqFILa |
| 6. | [HQ]EIP[RK][PM]PP[IV][NR][RP] | 21 | 11 | BrCRC |
|  |  |  |  | VvFILa, VvFILb, AtFIL, BrFILa, BrFILb, BrFILc, BsFIL, CqFILa |
|  |  |  |  | VvYAB2a, AtYAB2, BrYAB2a, BrYAB2b, BrYAB2c, BsYAB2, CqYAB2a,CqYAB2b |
|  |  |  |  | AtYAB3, BrYAB3, BsYAB3, CqFILa |
| 7. | [MV][MGL]MKEGF[YL]A[PS][AQ] | 11 | 11 | VvFILa, VvFILb, AtFIL, BrFILa, BrFILb, BrFILc, BsFIL, CqFILa |
|  |  |  |  | AtYAB3, BrYAB3, CqFILa |
| 8. | [QK][GQ][KQ][CQR]LD[QH][QT][VI][AST][GL][QE][GK][SFT]N | 12 | 15 | VvCRC, CqCRCa, CqCRCb, BsCRC, |
|  |  |  |  | BrINOa |
|  |  |  |  | AtYAB2, BsYAB2b, BrYAB2a, BrYAB2b, BrYAB2c, CqYAB2a, CqYAB2b |
| 9. | [ML][ML]KASF[VI]PLHL[LF][SAT][SA]L[SN][QH][DL][DQ][EV][KNT] | 7 | 21 | VvINO, AtINO, BrINOa, BrINOb, BsINO, CqINOa, CqINOb |
| 10. | [HQ][QS][ST][PS][AP][PT]PIHQDLQ[QP][HP][KR]Q[HQ][IT][TA][ST][SL][VI]TRK[ED][CHWY][GA]SSSRS[SFT]N[HN][FL]S[TE][TN] | 4 | 41 | AtYAB2, BrYAB2a, BrYAB2b, BrYAB2c |
| 11. | NM[RA][AS][LA][LF][LQ][PS][AS]S[NS]QL[QH][LQ] | 18 | 15 | VvFILa, VvFILb, AtFIL, BrFILa, BrFILb, BrFILc, BsFIL, CqFILa |
|  |  |  |  | VvYAB2a, VvYAB2b |
|  |  |  |  | AtYAB3, BrYAB3,BsYAB3, CqFILa |
|  |  |  |  | VvYAB5, CqYAB5a, CqYAB5b, BsYAB5 |
| 12. | [EK]E[VE][AV]A[TA]TDGVE[EK]EAWKV[NT]QEKENSP[TA]TLV[TS]SS[DE][NS]EDED | 3 | 38 | AtINO, BrINOa, BrINOb |
| 13. | [LF][EG]DH[ED][EV]SNNGFRERKAQRHSIWGKSPF[ED] | 3 | 28 | AtINO, BrINOa, BrINOb |
| 14. | SS[SM]SS[SP]S[ST][TA]V[AT][SE][PD][DV][HS] | 18 | 15 | VvCRC, AtCRC, BrCRC, CqCRCa, CqCRCb, BsCRC |
|  |  |  |  | AtFIL, BrFILa, BrFILb, BrFILc, BsFIL, CqFILa |
|  |  |  |  | BsYAB2, CqYAB2a, CqYAB2b |
|  |  |  |  | AtYAB3, BrYAB3, CqFILa |
| 15. | [MI]DRHSTTLSMLPSSEEDND[DY]E[ED]E[DE][ED]E[ED][DN] | 3 | 28 | BsINO, CqINOa, CqINOb |
| 16. | HQQQN[FY]HQA[PQ]N[NG]GNM[GN]EY[RK][IL][DE]NLGSSS | 3 | 27 | BsYAB5, CqYAB5a, CqYAB5b |
| 17. | HP[NT]MNDIPS[FL]M[DN][LI]HQ | 4 | 15 | AtFIL, BrFILa, BrFILb, BrFILc |
| 18. | [MT][FN]N[QD]P[KM][LM][DS][EV][GR][GS][VQ][DK]HL | 6 | 15 | BsFIL, CqFILa |
|  |  |  |  | CqFILa |
|  |  |  |  | VvYAB5, CqYAB5a, CqYBA5b |
| 19. | [NVT][IS][RSA][EVL][EQY][QR][IV][VI]N[KR]P | 10 | 11 | AtINO, BrINOa, BrINOb, BsINO, CqINOb, |
|  |  |  |  | VvYAB5, AtYAB5, BrYAB5, CqYAB5a, CqYAB5b |
| 20. | N[VI]GVS[PL] | 17 | 6 | VvFILa, VvFILb, AtFIL, BrFILa, BrFILb, BsFIL, CqFILa |
|  |  |  |  | AtYAB2, BrYAB2a, BrYAB2b, BrYAB2c, BsYAB2, CqYAB2a,CqYAB2b |
|  |  |  |  | AtYAB3, BrYAB3, CqFILa |
