# Supplementary material for: Comparative Analysis of the YABBY Gene Family of Bienertia sinuspersici, a Single-Cell C4 Plant
Source: Plants (Basel). 2019 Nov 22;8(12):536. doi: 10.3390/plants8120536 (PMC6963775; doi:10.3390/plants8120536)
Supplement: Supplementary file 1 [file plants-08-00536-s001.zip › spm-plants-619033/Supplementary Table_2.docx]

**Supplementary Table 2:** Primers used for RT-qPCR analysis

| **Gene name** | **Gene ID** | **Forwards primer** | **Reverse primer** | **Amplicon size** |
| --- | --- | --- | --- | --- |
| *AtCRC* | *NP_177078.1* | CTCCTCTTCAAGGCCATGTTA | GTGGAGGAAGAGGAAGAAGAAG | 96 |
| *BrCRC* | *XP_009105464.1* | CTCTTCTTCCTCCTCCTCTTCT | TGGGAGTCTCTGCTTCTTCT | 96 |
| *BsCRC* | *MK782018* | CTTCGTCATCATCCACCGTAAA | GTAAGCCGATGGAAGCCTATG | 92 |
| *AtINO* | *NP_001320962.1* | CTTCCTTCATTCCTCTCCATCTC | GCTTCTTCTTCCACACCATCT | 96 |
| *BrINOa* | *XP_009115528.1* | GTCTCATATGGATGAGAAGGGGC | GGTGGTTTGTTGACAACTTGGTAA | 175 |
| *BrINOb* | *XP_009103178.1* | CTCTCATCTTGATGAGACGGAAA | CTGAAGATGTAACCAAAGTCGC | 121 |
| *BsINO* | *MK782019* | CCTCCTTTGTTCCCTTTCATCT | CAATGTAGTGCTGTGCCTATCT | 113 |
| *AtFIL* | *NP_566037.1* | GAGAGTCCCATCCGCATATAAC | TCTTGGCAGCAGCACTAAA | 106 |
| *BrFILa* | *XP_009142321.1* | CCAGAGAACCTCTCACCTGA | GGCCGATCTCATGTTCACAG | 170 |
| *BrFILb* | *XP_009133711.1* | TCACATACACTTCGGACTCGCA | CACGGGTTGATTGTCTGGTGC | 40 |
| *BrFILc* | *XP_009104094.1* | GATTCCTAAAGCACCACCCACT | TCACGGGTTGATTGTCTGGCAT | 205 |
| *BsFIL* | *MK782020* | TAATCGCCCTCCAGAGAAGA | AGGCCTCCCTGTGACTAATA | 113 |
| *AtYAB2* | *NP_001077490.1* | GCTATCCCTCAACATTGGAGTT | GAGAGGTTGTGTGCTGTCTATG | 91 |
| *BrYAB2a* | *XP_009148101.1* | CACTTCATCAAAGCTCACCAGC | AGCTCCTAGAAGATGATCCGTG | 89 |
| *BrYAB2b* | *XP_009110891.1* | CAGCATAAGCAACAGATAACAA | GATGAATCTGTTGTAGGCT | 178 |
| *BrYAB2c* | *XP_009118372.1* | GTTTCACTTCATCAAAGCTCG | CTCCTGGACGATGATCCCC | 91 |
| *BsYAB2* | *MK782021* | TCTCCTCCACCTATCCCTAATC | GCCTGTAGTCATACTGCTATCC | 99 |
| *AtYAB3* | *NP_567154.1* | GGCTCATTTCCCTCACATACA | CTTCTCTTCCCATCATCCCATC | 106 |
| *BrYAB3* | *XP_009111512.1* | GGCTCATTTCCCTCACATACA | CTTCCCATCATCACTTCCTCTC | 101 |
| *BsYAB3* | *MK782022* | CAACTCTCACTATCATCGTCTGG | CGATGTCGGAGCCATGTAAT | 106 |
| *AtYAB5* | *NP_850080.1* | CCCTAATTTCCAGGCAACAAAC | TGCTCAGTTATGGTACGAGTTG | 104 |
| *BrYAB5* | *XP_009133935.1* | GCACCAATCTGTGGTCTGTA | GCCTCTTGAGGAAGATCCATAC | 109 |
| *BsYAB5* | *MK782023* | AGCGTGTACCATCTGCTTAC | TGAATGCTTCCCTGTGACTAAT | 93 |
| *AtGAPDH* | *NP_187062.1* | CATGACCACTGTCCACTCTATC | CACCAGTGCTGCTAGGAATAA | 116 |
| *BrGAPDH* | *Bra040213* | CCACCGTTGATGTCTCAGTT | GCCTTCAGATTCCTCCTTGATAG | 98 |
| *BsGAPDH* | *Bsv0100-00017536-RA* | CTCCAGGAATCCAGAGGAGATA | CTTTGCTCCACCCTTCAAATG | 118 |

*BsGAPDH* have not been submitted to NCBI yet. Therefore, gene identification number was given in the order number from total identified genes of *Bienertia sinuspersici* genome.
