# Supplementary material for: Comparative Analysis of the YABBY Gene Family of Bienertia sinuspersici, a Single-Cell C4 Plant
Source: Plants (Basel). 2019 Nov 22;8(12):536. doi: 10.3390/plants8120536 (PMC6963775; doi:10.3390/plants8120536)
Supplement: Supplementary file 1 [file plants-08-00536-s001.zip › spm-plants-619033/Supplementary Table_3.docx]

| **Stages** | ***At*_*GAPDH*** | | | **Average C_T_** | **SE** |
| --- | --- | --- | --- | --- | --- |
| Leaf | 18.650 | 18.740 | 18.850 | 18.747 | 0.058 |
| Final Bud | 21.440 | 21.440 | 21.440 | 21.440 | 0.000 |
| Preanthesis | 19.890 | 19.920 | 20.010 | 19.940 | 0.036 |
| Anthesis | 20.290 | 20.270 | 20.350 | 20.303 | 0.024 |
|  | | | | | |
| **Stages** | ***Br*_*GAPDH*** | | |  |  |
| Leaf | 20.110 | 19.890 | 20.050 | 20.017 | 0.066 |
| Final Bud | 19.000 | 19.090 | 19.080 | 19.057 | 0.028 |
| Preanthesis | 20.530 | 20.650 | 20.630 | 20.603 | 0.037 |
| Anthesis | 19.810 | 19.750 | 20.070 | 19.877 | 0.098 |
|  | | | | | |
| **Stages** | ***Bs*_*GAPDH*** | | |  |  |
| Leaf | 25.130 | 24.800 | 24.950 | 24.960 | 0.095 |
| Final Bud | 26.180 | 26.170 | 26.380 | 26.243 | 0.068 |
| Preanthesis | 23.590 | 23.530 | 23.760 | 23.627 | 0.069 |
| Anthesis | 24.320 | 24.150 | 24.370 | 24.280 | 0.067 |

**Supplementary Table 3:** Expression of *GAPDH* in threshold cycle (C_T_) value between different tissue types studied in *Arabidopsis thaliana* (*At*), *Brassica rapa* (*Br*), and *Bienertia sinuspersici* (*Bs*).
