# Supplementary figures and images for: Comparative Analysis of the YABBY Gene Family of Bienertia sinuspersici, a Single-Cell C4 Plant
Source: Plants (Basel). 2019 Nov 22;8(12):536. doi: 10.3390/plants8120536 (PMC6963775; doi:10.3390/plants8120536)

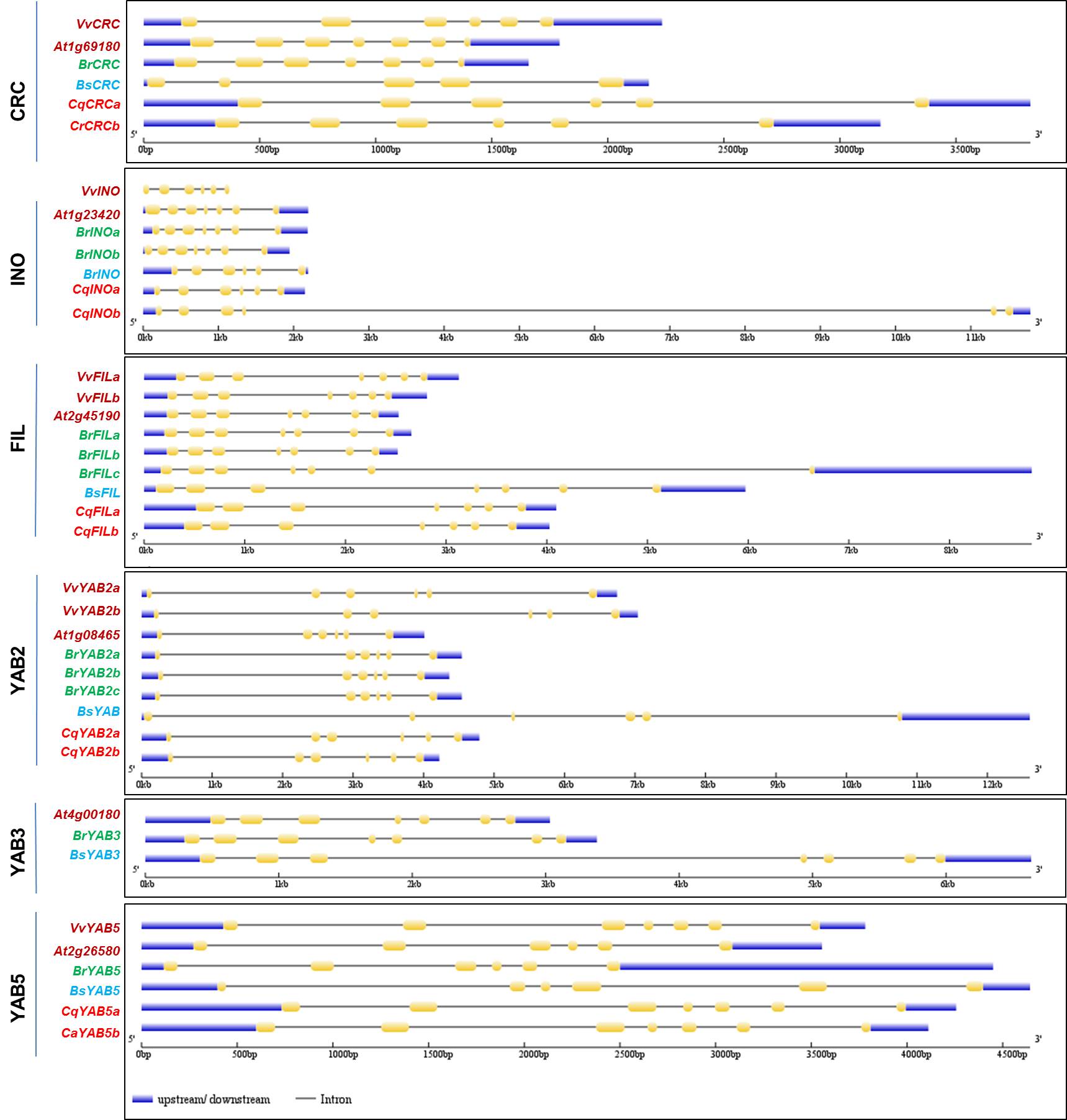

Supplement: Supplementary file 1 [file plants-08-00536-s001.zip › spm-plants-619033/Supplementary Fig_1.jpg]

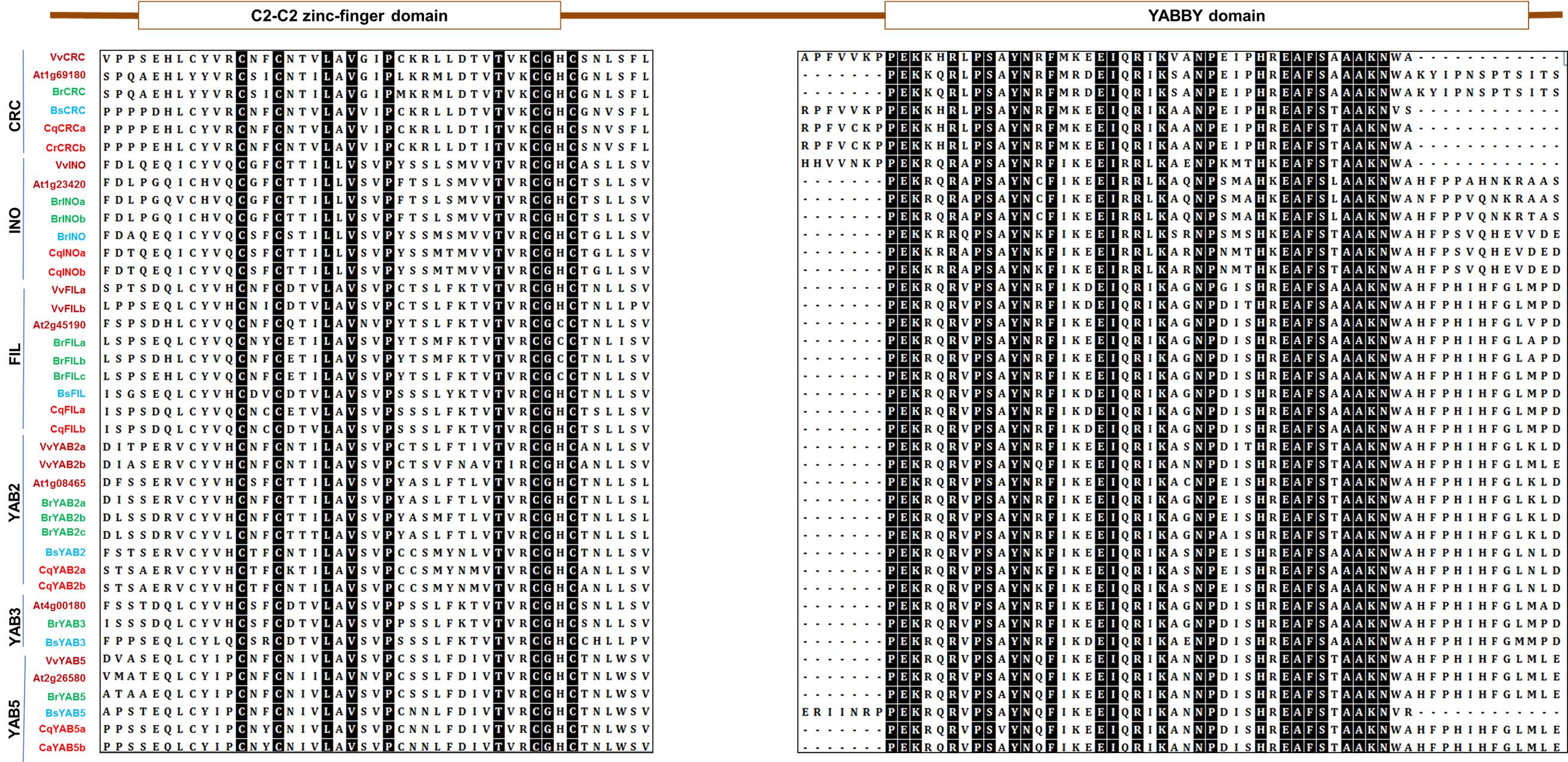

Supplement: Supplementary file 1 [file plants-08-00536-s001.zip › spm-plants-619033/Supplementary Fig_2.jpg]
